# Tumor-Associated Tertiary Lymphoid Structures: Gene-Expression Profiling and Their Bioengineering

**DOI:** 10.3389/fimmu.2017.00767

**Published:** 2017-06-30

**Authors:** Genyuan Zhu, Rana Falahat, Kui Wang, Adam Mailloux, Natalie Artzi, James J. Mulé

**Affiliations:** ^1^Immunology Department, Moffitt Cancer Center, Tampa, FL, United States; ^2^Department of Medicine, Division of Engineering in Medicine, Brigham and Women’s Hospital, Harvard Medical School, Boston, MA, United States; ^3^Harvard-MIT Division of Health Sciences and Technology, Institute for Medical Engineering and Science, Massachusetts Institute of Technology, Cambridge, MA, United States; ^4^Cutaneous Oncology Department, Moffitt Cancer Center, Tampa, FL, United States

**Keywords:** chemokines, scaffolds, bioengineering, immune cells, antitumor

## Abstract

Tertiary lymphoid structures (TLSs) have been identified in the parenchyma and/or in the peripheral margins of human solid tumors. Uncovering the functional nature of these structures is the subject of much intensive investigation. Studies have shown a direct correlation of the presence of human tumor-localized TLS and better patient outcome (e.g., increase in overall survival) in certain solid tumor histologies, but not all. We had identified a tumor-derived immune gene-expression signature, encoding 12 distinct chemokines, which could reliably identify the presence of TLSs, of different degrees, in various human solid tumors. We are focused on understanding the influence of TLSs on the tumor microenvironment and leveraging this understanding to both manipulate the antitumor immune response and potentially enhance immunotherapy applications. Moreover, as not all human solid tumors show the presence of these lymphoid structures, we are embarking on bioengineering approaches to design and build “designer” TLSs to address, and potentially overcome, an unmet medical need in cancer patients whose tumors lack such lymphoid structures.

Despite the clinical success with antibodies against CTLA-4 and PD-1, cytokines (e.g., high-dose interleukin-2), as well as the adoptive transfer of tumor-infiltrating lymphocytes (TILs), many patients treated with those agents fail to respond in a clinically meaningful manner. Employing Moffitt Cancer Center’s revolutionary Total Cancer Care (TCC) bio-repository (>38,000 tumors), genomic database (>16,000 tumor gene-expression arrays; ~5,000 tumor whole genome, whole exome, and targeted gene sequences), and longitudinal clinical database (on >100,000 TCC consented patients), we identified a unique 12-chemokine (CCL2, CCL3, CCL4, CCL5, CCL8, CCL18, CCL19, CCL21, CXCL9, CXCL10, CXCL11, and CXCL13) gene-expression signature (GES) from a metagene grouping with overwhelming enrichment for immune-related and inflammation-related genes. The GES was interrogated on 14,492 distinct solid tumors of 24 distinct tissue types (primaries and metastases) in TCC (modified Affy chip) and confirmed on another set of >7,000 samples in the TCGA database (RNASeq) and showed distribution across different histologies, including breast, lung, melanoma, and colorectal cancers of differing degrees (1–4). We showed that this 12-chemokine GES could accurately predict the degree and type of lymphoid infiltrate, organized remarkably as tumor-localized, tertiary lymphoid structures (TL-TLSs) that comprise—by immunohistochemistry staining—prominent B cell follicles, T cell marginal zones, and associated follicular dendritic cells (DCs) but few, if any, T regulatory cells (2–4). TL-TLSs appear to exhibit similar structural and cellular characteristics of peripheral lymph nodes and presumably arise in the tumor microenvironment in response to chronic inflammation (5–7).

Of importance, there was a highly significant and consistent association between a marked increase in overall patient survival, the value of the mean score of the GES, and the presence of TL-TLSs in stage IV (non-locoregional) melanoma, colorectal cancer, and, most recently, in stage IV bladder cancer, non-small cell lung cancer, and certain types of breast cancer, especially in basal and HER2^+^ patients. Moreover, we have now demonstrated that solid tumor radiosensitivity across a spectrum of histologies is associated with immune activation as measured by the GES^1^ (8). It remains unclear whether TLSs are either only a consequence of an immune response *per se* or sites of an active immune reaction against the local tissue components. With respect to the latter, previous studies showed that TLSs are associated with longer survival of cancer patients, aggravation of graft rejection, and presence of auto-immune inflammatory disease (9). TLSs have also been observed as a consequence of immunization with certain vaccines. As examples, intramuscular vaccination targeting HPV16 in patients with cervical intraepithelial neoplasia induced cervical tissue immune infiltrates, including organized TLSs (10). In a pancreatic ductal adenocarcinoma clinical trial, an irradiated, allogeneic granulocyte-macrophage colony-stimulating factor-secreting pancreatic tumor cell vaccine converted an “non-immunogenic” neoplasm into an “immunogenic” one by inducing formation of TLSs (11). Gene-expression analysis of the vaccine-induced TLSs showed a suppressed Treg pathway and an enhanced Th17 pathway, which was associated with improved patient survival and elicitation of mesothelin-specific T-cell responses. In another study in mice with deficiency of secondary lymphoid organs, infection of influenza virus could stimulate neogenesis of lung TLSs that produced an efficient protective immune response (12). Collectively, these studies argue indirectly in favor of an active participatory role of TLSs in effective immune responses. Therefore, local induction of TLSs in the tumor microenvironment could be a promising therapeutic strategy to exploit against cancer.

We believe this unique GES may potentially be used for preselecting cancer patients for broad immunotherapy interventions (e.g., vaccines, cytokines, and/or immunoregulatory antibodies) to increase clinical response rates by identifying the presence of antitumor reactive, TLSs existing within tumor masses. Clinical trials in melanoma patients are pending to test this hypothesis. In addition, this GES is currently being used to potentially identify solid tumor masses, beyond melanoma, capable of providing effective TILs for *ex vivo* expansion for adoptive transfer into patients (13). The existence of a functional connection between TL-TLSs and identifying (and expanding) tumor-specific, therapeutic TIL is underway. Finally, in collaboration with Eli Pikarsky at the Hadassah Medical Center, we are investigating why patients with certain GES-positive solid tumors with TL-TLSs (i.e., hepatocellular carcinoma) actually show poor prognosis, suggesting that not all TL-TLSs are beneficial.

The 12-chemokine GES has now also provided the actual gene leads for potentially constructing bioengineered “designer lymph nodes.” The novel platform is based on the improvement, manipulation, and stimulation of the host’s own immune system. We are using a specialized antigen-presenting DC, produced from the host’s blood or bone marrow, which is both antigen(s) loaded and genetically manipulated to express highly selected chemokine genes combined with biomaterial scaffolds prior to administration into cancer-bearing hosts (14). This gene-modified cell-scaffold platform “design builds” a functioning “lymph node” on its own at any administration site that then produces a preplanned immunologic response against cancer cells locally and then throughout the host’s body. The technology includes the option of providing gene-modified cell-scaffold platforms at multiple, independent sites to create multiple, independent “lymph nodes” of the same function and specificity concurrently. The administrations can also be staggered to create additional new structures over time. Moreover, by administering pools of different gene-modified cell-scaffold platforms, we hypothesize that these structures will act independently of each other and will create distinct functioning “lymph nodes” in the same host. Utilized by the host, these “designer lymph nodes” can provide an enhanced, unified, or diversified immune system to fight cancer.

Tertiary lymphoid structures have been described in animal models. Previous studies on gene knockout mice have identified the role of lymphotoxin (LT) in development of lymphoid organs (15). LTα-deficient mice, which lack the soluble LTα3 homotrimer as well as the membrane LTα1β2 heterotrimer, show absence of Peyer’s patches and all peripheral lymph nodes (16, 17). LTβ-deficient mice also displayed similar defect with residual mesenteric and cervical lymph nodes (18). Consistently, transgenic mouse models that ectopically express LTα or LTβ demonstrate formation of TLSs in non-lymphoid tissues. For instance, restricted expression of LTα or LTα/LTβ in kidney and pancreas induced organized infiltrates in these sites that show similar cellular composition to lymphoid organs. T cells, B cells, plasma cells, antigen-presenting cells, and features of high-endothelial venules (HEV) were observed in the infiltrates (19, 20). These studies suggest that the formation of TLSs involves the same signaling pathways in development of the secondary lymphoid organs.

In addition to the LT pathway, several transgenic mouse models with overexpression of chemokines that are important for recruiting immune cells displayed TLSs. In a transgenic mouse model with pancreatic islet-specific expression of CCL21, spontaneous development and organization of lymphoid tissues composed of T cells, DC, B cells, HEV, and stroma reticulum was observed (21, 22). Similarly, constitutive expression of CCL21 in thyroid resulted in significant lymphocytic infiltrates, which are topologically arranged into B and T cell areas (23). In addition to CCL21, ectopic expression of another three important homeostatic chemokines CCL19, CXCL12, and CXCL13 in pancreatic islet led to formation of TLSs with different size, cellular composition, and organization (24, 25). Moreover, in a recent study, transplantable and functional “artificial” TLSs could be constructed from slow-releasing gels containing different lymphogenesis-related chemokines and ligands (26). Collectively, these studies indicated that the formation of TLSs could be achieved by chemokine-mediated accumulation of lymphocytes.

Sound preclinical models of TLS formation are being developed in which multiple elements are being interrogated for the capacity to recruit and design an antitumor immune system. The inclusion of lymph node-derived primary cellular components, which normally provide chemotactic and homeostatic cues in conventional lymph nodes (27), are being genetically modified to express selected chemotactic and lymphoid neogenesis-related genes to enhance TLS formation. Previous studies have suggested that inclusion of activated DCs in a stromal cell-induced TLS model could markedly enhance the efficiency and organization of TLS formation (28). The contributions of DCs to TLS induction have been reviewed elsewhere (29). In this regard, CD11c^+^ DCs were necessary for maintenance of inducible bronchus-associated lymphoid tissues. Additionally, retinoic acid production by DCs from gut-associated lymphoid organs was involved in the imprinting of gut-homing receptors on activated T cells. Various modified cell lines are being combined with tumor antigen-pulsed DCs and then incorporated in biocompatible scaffold materials and administered to tumor-bearing mice as injectable or implantable matrices. These matrices should serve as model systems to better understand the factors governing the formation and/or maintenance of TLSs and also to identify and classify tumor-specific, therapeutic TIL. Additionally, these matrix-based systems may function as a therapeutic platform by delivering, stimulating, and expanding transplanted lymphocytes and/or modified DCs.

Scaffolds are typically three-dimensional microporous structures designed to enable *ex vivo* cell encapsulation and/or *in vivo* cell infiltration while providing mechanical support, cell adhesion, and a sustained supply of biological cues to promote cell migration and interactions (30, 31). The scaffolds must be biocompatibile and should maintain a robust state for adequate time to allow the development of a new tissue, while eventually undergoing degradation and be replaced by the new tissue. Based on the origin of materials, scaffolds can be classified into synthetic or natural polymeric systems (32). We have developed optical means by which to track the fate of implanted materials (33) and embedded cells fate (34) non-invasively over time. This enables us to study cell:material interactions *in vivo* and to rationally modify the materials, as needed, to attain the desired preclinical outcomes.

Synthetic polymers can be readily tailored and modified to provide excellent mechanical and chemical properties; however, they typically lack essential biological elements that are required to induce necessary cell responses. For example, poly(lactic acid), poly(glycolic acid), and poly(caprolactone) are the most widely used synthetic biodegradable polymers, but their hydrophobic nature limits their application in tissue engineering due to the insufficient water absorption, cell adhesion, and interactions. In contrast, hydrophilic synthetic polymers such as poly(ethylene glycol) (PEG) and poly(ethylene oxide) possess excellent solubility in a wide range of solvents. However, they are not biologically degradable; therefore, they can only be used as permanent implants or as low-molecular weight blocks in combination with other components to enable elimination from the body (35).

Natural polymers are particularly attractive candidates for tissue-engineering applications, as they offer excellent biocompatibility. Unlike synthetic polymers, they can be biologically degraded into components, which are non-inflammatory or non-toxic. The reactive sites available in most of the natural polymers allow ligand conjugation, cross-linking, and other modifications to tune their properties for specific applications (36). Protein-based natural polymers such as collagen and gelatin have the potential to control the cellular migration, proliferation, and organization during new tissue development, as they possess many features of extracellular matrix. However, they often suffer from batch-to-batch variations due to the difficulties associated with the purification processes. Polysaccharides (alginate, chitosan, starch, and hyaluronic acid derivatives) are another promising class of natural and biocompatible polymers. Chitosan in particular is an important example with an established safety profile in humans (37, 38).

In addition, a diverse collection of hybrid scaffolds using different combinations of synthetic and natural polymers has been developed for specific tissue-engineering applications. As examples, a polyurethane scaffold containing type I collagen and matrigel was used to create 3D lymph node T zone stromal models (39). A different hybrid scaffold approach incorporating a collagen matrix into PEG hydrogel was used by Stachowiak and Irvine to improve the migration of loaded T cells and DCs within the framework (40). When needed, scaffolds can be doped with microparticles or nanoparticles for controlled release of soluble factors in addition to serving as a scaffold for cells. Such measures can provide sustained environmental cues to augment antigen-presenting DCs or lymphocyte longevity, maturation, and activation. Hence, synthetic materials can be modified to include biological cues and harbor sites for cells to serve as engineered scaffolds, which can be manufactured in a reproducible manner and with controlled properties. Hydrogels, in particular, can provide a controlled cell microenvironment for immune cells that enable the recruitment, expansion, and activation of immune cells *ex vivo* and *in vivo* (41). The choice of materials is dictated by the end use, including biocompatibility, immunogenicity, site of implantation, types of stimuli, and release kinetics. Hydrogels have been utilized in both active and passive immunotherapies. They can be used to deliver antigens, chemokines, and other factors to DCs, to induce T cell stimulation and B cell-mediated antibody responses, or they can allow for efficient encapsulation of immunomodulatory molecules as well as immune cells. Immune cells such as DCs can be activated *ex vivo* in hydrogels prior to their implantation or *in vivo* by immobilizing stimuli within the gels that would recruit and activate the cells inside the gels, as bioreactors. Localized administration of the immunomodulatory hydrogels next to the tumor site offers higher bioavailability and controlled release of embedded molecules or of factors from embedded/recruited cells, over time. In particular, we have exploited injectable hydrogel-based adhesive scaffolds that can adhere to the surface of a tumor to locally release molecules of interest to combat cancer (42–45).

Future clinical trials will plan to incorporate many/all of the 12 chemokines identified by the molecular signature coupled with appropriate biomaterial scaffolds in an attempt to achieve the “optimal” lymph node design in immunosuppressed cancer patients.

## Author Contributions

All authors contributed to the preparation of this manuscript.

## Conflict of Interest Statement

The authors declare that the research was conducted in the absence of any commercial or financial relationships that could be construed as a potential conflict of interest.

## Footnotes

^1^Strom T, Harrison LB, Giuliano A, Eschrich SA, Berglund A, Fulp W, et al. Tumor radiosensitivity is associated with tumor-directed immune activation as measured by two gene expression signatures. (Forthcoming).
